# UNMET CAREGIVING NEEDS AMONG HOME HEALTH PATIENTS WITH DEMENTIA: FRONTLINE CLINICIAN REPORTS

**DOI:** 10.1093/geroni/igae098.1233

**Published:** 2024-12-31

**Authors:** Julia Burgdorf

**Affiliations:** VNS Health, New York, New York, United States

## Abstract

One-third of Medicare home health care (HHC) patients have diagnosed dementia and rely heavily on support from family caregivers to implement the plan of care, but no prior work has examined unmet caregiving needs among these patients. Drawing on clinician reports in 2018 Medicare HHC assessment data for a national sample of 426,606 HHC patients with diagnosed dementia, we define unmet caregiving needs stemming from lack of availability (i.e., no caregiver is present) or lack of capacity (i.e., caregiver requires clinician supervision and training). Given the progressive nature of the disease, we compare across 3 levels of dementia symptom severity, determined by clinician-reported cognitive and behavioral symptoms. Comparing those with mild, moderate, and severe dementia symptoms, unmet needs due to lack of caregiver availability were present for 9.1%, 6.9%, and 5.3% of patients, respectively; unmet needs due to lack of caregiver capacity were present for 70.6%, 71.1%, and 74.9% of patients, respectively. In adjusted multilevel models controlling for patient and HHC agency characteristics and clustering at the agency level, individuals with more severe dementia symptoms were less likely to have an unmet need for caregiver assistance due to a lack of caregiver availability (aOR: 0.63; 95% CI: 0.60-0.66), but more likely to have an unmet need due to a lack of caregiver capacity (aOR: 1.15; 95% CI: 1.12-1.17). Findings suggest the need for varied approaches to caregiving-related support based on dementia disease stage and highlight widespread needs for clinicians to provide caregiver training during HHC episodes for those with dementia.

